# Isolation and molecular characterization of *Salmonella* spp. from chevon and chicken meat collected from different districts of Chhattisgarh, India

**DOI:** 10.14202/vetworld.2015.702-706

**Published:** 2015-06-06

**Authors:** V. K. Naik, S. Shakya, A. Patyal, N. E. Gade, Bhoomika

**Affiliations:** 1 Department of Veterinary Public Health and Epidemiology, College of Veterinary Science and Animal Husbandry, Chhattisgarh Kamdhenu Vishwa Vidyalaya, Anjora, Durg, Chhattisgarh, India; 2 Department of Veterinary Physiology and Biochemistry, College of Veterinary Science and Animal Husbandry, Chhattisgarh Kamdhenu Vishwa Vidyalaya, Anjora, Durg, Chhattisgarh, India

**Keywords:** chevon, Chhattisgarh, chicken, isolation, molecular characterization, *Salmonella*

## Abstract

**Aim:**

The aim was to assess the prevalence of *Salmonella* in raw chevon and chicken meat sold in the retail meat shops situated in and around Durg, Rajnandgaon, Dhamtari, Raipur, and Bilaspur districts of Chhattisgarh. Studies were also conducted to find out the antibiotic resistance in *Salmonella* isolates.

**Materials and Methods:**

A total of 400 samples comprising of 200 chevon meat and 200 chicken meat samples were processed for isolation of *Salmonella* and all isolates were further confirmed on the basis of cultural and biochemical characters and by targeting *inv*A gene of Salmonella. All *Salmonella* isolates were also examined for their antimicrobial drug susceptibility/resistance pattern against commonly used antibiotics.

**Results:**

Out of 400 samples, the prevalence of *Salmonella* in chevon and chicken meat was found 9% and 7% respectively, with an overall prevalence of 8%. Polymerase chain reaction targeting *inv*A gene of *Salmonella* showed positive result with 31 isolates. All 32 *Salmonella* isolates were found to be highly sensitive to ciprofloxacin while 96.87%, 96.87% and 93.75% were sensitive to gentamicin, imipenem, and ceftazidime, respectively. 93.75% and 59.37% isolates were resistant to erythromycin and oxytetracycline, respectively. Out of 32, 14 isolates had multiple antibiotic resistance index equal to or more than 0.2.

**Conclusion:**

*Salmonella* in chevon and chicken meat samples is prevailing in the areas of sampling due to poor hygienic conditions and also demonstrated the varied spectrum of antimicrobial resistance, including several multiple drug resistance phenotypes. Therefore, the present study emphasizes the need for continued surveillance of zoonotic foodborne pathogens including antimicrobial-resistant variants throughout the food production chain.

## Introduction

Among all foodborne diseases, *Salmonella* is identified as a leading cause of foodborne illness in humans and animals resulting in 16 million annual cases of typhoid fever, 1.3 billion cases of gastroenteritis and 3 million deaths worldwide [1]. In India, salmonellosis is endemic and its importance as potential zoonosis needs no emphasis as it causes heavy economic losses every year [2]. Outbreaks due to *Salmonella* have been associated with a wide variety of foods especially those of animal origin [3] such as meat, chicken, egg, and sometimes vegetables in the food chain [4,5].

The standard conventional cultural techniques to identify *Salmonella* spp. are time-consuming and can require up to 7 days for confirmation. Polymerase chain reaction (PCR) based methods combine simplicity with a potential for high specificity and sensitivity in detection of *Salmonella*. Amplification of *invA* gene of *Salmonella* has been reported as a suitable target for PCR amplification, with potential diagnostic applications [6]. During past two decades, antibiotic-resistant *Salmonella* has emerged and become a serious public health issue worldwide [7]. Currently, there are several evidences of therapeutic failure due to the increasing incidence of antimicrobial resistance among *Salmonella* species [8].

Therefore, the present study was conducted to assess the prevalence of *Salmonella* in chevon and chicken meat collected from five districts of Chhattisgarh, India. Studies were also conducted to find out the antibiotic resistance in *Salmonella* isolates.

## Materials and Methods

### Ethical approval

Live animals were not used in this study, so ethical approval was not necessary. Meat samples were collected from retail meat shops.

### Sample collection

A total of 400 samples comprising Chevon meat (n=200) and Chicken meat (n=200) were collected from retail meat shops situated in and around Durg, Rajnandgaon, Dhamtari, Raipur and Bilaspur districts of Chhattisgarh randomly during September 2013 to August 2014. All meat samples were aseptically collected in sterile polythene bags and transported in refrigerated conditions to the laboratory as soon as possible and processed within 5 h for bacteriological examination.

### Isolation and biochemical characterization

Isolation of *Salmonella* spp. from chevon and chicken meat samples was carried out as per the ISO 6579:2002 protocol [9] with slight modifications. Briefly, for pre-enrichment 25 g of meat sample was blended and discharged in 225 ml of sterilized buffered peptone water (BPW) (HiMedia, India) and incubated at 37°C for 20-24 h. One ml of culture from BPW broth was inoculated into the tube containing 10 ml sterile tetrathionate broth (TT) (HiMedia, India) for selective enrichment and further incubated at 37°C for 24 h. A loop full culture was then picked from TT broth and streaked onto the multiple selective plating media i.e. brilliant green agar (BGA) and bismuth sulfite agar (BSA) (HiMedia, India). All the inoculated plates were incubated at 37°C for 24 h. Moderately large, moist, smooth, and colorless colonies with pink background on BGA and typical black colony surrounded by brownish-black zone with metallic sheen on BSA were considered as *Salmonella*. The characteristic colonies of *Salmonella* spp. from selective plating media were further streaked on differential plating media, MacConkey’s lactose agar (HiMedia, India), and incubated at 37°C for 24 h. Characteristic colorless colonies of *Salmonella* were transferred to nutrient agar slant for further identification and biochemical characterization. All *Salmonella* isolates were biochemically tested using indole (I), methyl red (M), *voges proskauer* (Vi), citrate (C), triple sugar iron (TSI), and urease tests as per the protocol described by Ewing [10]. The colonies showing *Salmonella* specific IMViC pattern (-+-+) were further inoculated on TSI slants and colonies producing alkaline slant (pink) and acidic butt (yellow) with or without H_2_S production (blackening) were tested for urease production on urea agar slants. All the urease negative isolates were considered as biochemically confirmed *Salmonella* isolates.

### Molecular characterization

All biochemically confirmed *Salmonella* isolates were further confirmed by targeting *invA* gene. For PCR, template DNA was isolated by boiling and snap chill method as outlined by Nagappa *et al*. [11]. Purity and concentration of DNA were detected by 0.8% agarose gel electrophoresis. Recommended primer set of forward primer (26 bp): 5’- GTGAAATTATCGCCACGTTCGGGCAA-3’ and reverse primer (22 bp): 5’- TCATCGCACCGTCAAAGGAACC-3’ were used to obtain a predicted product size of 284 bp [12]. Primers of *invA* gene used in this study were synthesized from Imperial Life Sciences (P) Limited, Gurgaon, Haryana, India. PCR was standardized following the protocol of Rahn *et al*. [12] with some modifications using the thermocycler (Mastercycler, Eppendorf, Germany). Briefly, in a reaction mixture, 2.5 μl of ×10 taq buffer, 1.5 mM MgCl_2_, 50 μM of each deoxyribonucleotide triphosphate, 10 pmol of each primers, 1 U Taq polymerase, 3 μl of template DNA, and nuclease free water to make the total volume 25 μl were used. PCR-cycling was performed with initial denaturation of 94°C for 5 min, followed by 30 cycles of denaturation at 94°C for 1 min, annealing at 55°C for 1 min, and extension at 72°C for 1 min. Final extension was done at 72°C for 5 min. After the completion of reaction cycles, the PCR product was electrophoresed on 1.5% agarose gel stained with ethidium bromide (0.5 μg/ml), analyzed under ultraviolet transilluminator (Biometra) and photographed under Gel Documentation System (Gel Doc™ XR, Biorad, USA). All the biologicals used during the present study were procured from Thermo Scientific (USA), Genetix (India), and Bangalore Genei (India).

### Antibiotic sensitivity testing

All *Salmonella* isolates were examined for their antimicrobial drug susceptibility/resistance pattern by disc diffusion technique [13]. Antibiotic discs impregnated with oxytetracycline (O, 30 μg), amoxycillin (AX, 10 μg), cephalexin (CN, 30 μg), ciprofloxacin (CIP, 5 μg), gentamicin (GEN, 30 μg), erythromycin (E, 10 μg), cefotaxime (CTX, 10 μg), nalidixic acid (NA, 30 μg), ampicillin (AMP, 10 μg), ceftazidime (CAZ, 30 μg), imipenem (IPM, 10 μg), amoxyclav (AMC, 30 μg), cefixime (CFM, 5 μg), and meropenem (MRP, 10 μg) (HiMedia, India) were used. The diameter of the zones of complete inhibition was measured and compared with the zone size interpretation chart provided by the supplier and were graded as sensitive, intermediate, and resistant. The multiple antibiotic resistance (MAR) index was also calculated for all *Salmonella* isolates following the protocol described by Krumperman [14], by applying formula a/b where “a” is the number of antibiotics to which an isolate was resistant and “b” is the number of antibiotics to which the isolates were exposed.

## Results and Discussion

Based on the cultural characteristics and biochemical characterization, a total of 32 presumptive *Salmonella* isolates were isolated (Table-1), with an overall prevalence of 8%. In chevon meat samples, highest prevalence of *Salmonella* was recorded in Durg district (12.5%), followed by Raipur and Dhamtari (10%), Rajnandgaon (7.5%), and Bilaspur (5%) districts. In chicken meat samples, highest prevalence of *Salmonella* was seen in Rajnandgaon (12.5%) district followed by Raipur and Bilaspur (7.5%), Durg (5%), and Dhamtari (2.5%) districts.

**Table-1:** Prevalence of *Salmonella* in chevon and chicken meat samples collected from different districts of Chhattisgarh.

District	Chevon meat			Chicken meat		
	
	Number of samples processed	Number of isolates recovered	Prevalence (%)	Number of samples processed	Number of isolates recovered	Prevalence (%)
Durg	40	5	12.5	40	2	5
Rajnandgaon	40	3	7.5	40	5	12.5
Raipur	40	4	10	40	3	7.5
Dhamtari	40	4	10	40	1	2.5
Bilaspur	40	2	5	40	3	7.5
Total	200	18	9	200	14	7

The overall prevalence of *Salmonella* in chevon meat was found 9% which corroborates with the previous reports [15,16]. However, higher prevalence rates of 13.88% [17], 14.20% [18], and 38.33% [19] were reported from different regions. On the contrary, lower prevalence rates of 1.25% [20], 2.5% [21], and 3.3% [22] were also reported in chevon meat by several investigators. In case of chicken meat, 7% prevalence of *Salmonella* was recorded; this is in accordance with earlier reports [17,23-25]. However, lower prevalence rate of 0.94% [26], 1.5% [27], and 4% [28] were reported by others. On the contrary, higher prevalence rate of 23.7% [29], 28.33% [19], 30% [30], 31.99% [31], and 44% [32] were observed in chicken meat samples in earlier findings. Differences in prevalence rates of *Salmonella* spp. from chevon and chicken meat samples in various studies may be attributed to multiple factors, such as geographic and seasonal variation, variations in sampling procedures and sample size, animal management practices, hygienic conditions during production and processing of meat and meat products or due to differences in the sensitivity and specificity of different isolation methods used.

**Figure-1:**
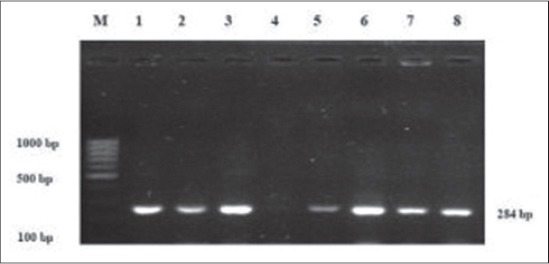
Agarose gel electrophoresis showing amplified polymerase chain reaction product of *inv*A gene. Lane M: 100 bp DNA Ladder, Lane 1-3 and 5-8: *Salmonella* isolates with *inv*A positive amplicons (284 bp), Lane 4: Negative control (No Template DNA added).

Molecular characterization of biochemically confirmed *Salmonella* isolates exhibit the desirable PCR product of 284 bp size of *invA* gene (Figure-1). Presence of *invA* gene confirms the invasive strains of *Salmonella* at the genus level. During the study, *invA* gene was amplified from 31 *Salmonella* isolates. The results of our study are in agreement to earlier findings [29,33,34]. It is speculated that strains without *invA* gene are not invasive, or that they might be using other invasive mechanisms. However, their absence in *Salmonella* seems to be rare [6].

All 32 *Salmonella* isolates were found to be highly sensitive to ciprofloxacin while 96.87%, 96.87% and 93.75% were sensitive to gentamicin, imipenem, and ceftazidime, respectively. The 93.75% and 59.37% isolates were resistant to erythromycin and oxytetracycline, respectively. Varying degree of sensitivity was found against amoxyclav, cefixime (81.25% each), nalidixic acid, amoxicillin and cephalexin (78.12% each), ampicillin (75%), and cefotaxime (59.37%). Sample wise antibiogram study revealed that all the isolates from chicken meat samples were 100% resistant to erythromycin whereas 88.88% of chevon meat isolates were resistant to erythromycin. In case of oxytetracycline, the order of resistance in chevon meat samples was (72.22%) followed by chicken meat (42.85%). Highest MAR index was 0.50 (one isolate) followed by 0.42 (one isolate), 0.35 (one isolate), 0.28 (three isolates), 0.21 (eight isolates), 0.10 (nine isolates), and 0.07 (eight isolates). The minimum MAR index 0 was shown by one isolates. Out of 32, 14 isolates were found to have MAR index equal to or more than 0.2, thus indicated the injudicious use of antibiotics. Similarly Jaulkar *et al*. [35] also reported MAR index ranging from 0.06 to 0.53 and found 10 out of 11 isolates are having MAR index more than 0.2. Hence, the present study reveals that *Salmonella* isolates from chevon and chicken meat are resistant to more than one antibiotic indicating the prevalence of multidrug resistant *Salmonella* in foods of animal origin. Findings of our study are in accordance with previous reports [29,36].

## Conclusion

Prevalence of *Salmonella* in chevon meat samples was found 9% whereas 7% in chicken meat samples in Durg, Raipur, Dhamtari, Rajnandgaon, and Bilaspur Districts of Chhattisgarh, pose a major threat for spread of Salmonellosis in humans. *Salmonella* needs special concern because of poor hygienic conditions prevailing in the areas of sampling which ultimately favors its spread. The isolates also exhibit the presence of *invA* gene thus confirms their invasiveness. The result of the study also demonstrated the varied spectrum of antimicrobial resistance, including several multiple drug resistance phenotypes among *Salmonella* isolates. Overall, antimicrobial resistance phenotypes were similar between *Salmonella* isolates recovered from chevon and chicken meat samples. This highlights the need for continued surveillance of zoonotic foodborne pathogens including antimicrobial-resistant variants throughout the food production chain.

## Authors’ Contributions

SS designed and planned this research work. VKN collected the samples and executed the isolation, biochemical, molecular characterization work. VKN and B carried out the antibiotic sensitivity assay of all isolates. AP analyzed the data and monitored the isolation, biochemical characterization and antibiotic sensitivity assay. NEG was involved in the molecular characterization experiment. All authors contributed equally in preparation and revision of the manuscript. All authors read and approved the final manuscript.
